# 25(OH)Vitamin D and autism spectrum disorder: genetic overlap and causality

**DOI:** 10.1186/s12263-023-00727-0

**Published:** 2023-04-26

**Authors:** GuoSheng Yu, MinZhi Xu, Yao Chen, HaiYan Ke

**Affiliations:** 1 Department of Pediatrics, Li shui People’s Hospital, The Sixth Affiliated Hospital, Wenzhou Medical University, Li shui, Zhejiang, 323000 China; 2 Department of Pediatrics, Tongde hospital of Zhejiang Province, 234 Gucui Road, Xihu District, Hangzhou City, 310006 China

**Keywords:** 25(OH)D, Autism spectrum disorder (ASD), Mendelian randomization analysis, Genome-wide association study

## Abstract

**Objective:**

To identify whether there exists a genetic correlation and causal relationship between 25(OH)D and autism spectrum disorder (ASD).

**Methods:**

Based on large-scale genome-wide association studies, a series of genetic approaches were adopted to obtain summary statistics. Using linkage disequilibrium score regression, we assessed the shared polygenic structure between traits and performed pleiotropic analysis under composite null hypothesis (PLACO) to identify pleiotropic loci between complex traits. A bidirectional Mendelian randomization (MR) analysis was applied to investigate whether there is a causal relationship between 25(OH)D and ASD.

**Results:**

The linkage disequilibrium score regression (LDSC) showed a negative genetic correlation between 25(OH)D and ASD (*r*_g_ = − 0.227, *P <* 0.05), and PLACO analysis identified 20 independent pleiotropic loci matched to 24 pleiotropic genes, of which the function reveals an underlying mechanism on 25(OH)D and ASD. In Mendelian randomization analysis, the inverse variance-weighted (IVW) method with OR = 0.941 (0.796, 1.112) and *p* < 0.474 did not show a causal relationship between 25(OH)D and ASD, while, in the reverse Mendelian randomization analysis, IVW method showed OR = 1.042 (0.930, 1.169), indicating no causal relationship either.

**Conclusion:**

This study provides evidence for a shared genetic overlap between 25(OH)D and ASD. Bidirectional MR analysis also did not show a definite causal relationship between 25(OH)D and ASD.

**Supplementary Information:**

The online version contains supplementary material available at 10.1186/s12263-023-00727-0.

## Introduction

Autism spectrum disorder (ASD), a group of neurodevelopmental disorders that begin in infancy, is characterized by a lack of social communication and interaction, stereotypical patterns of behavior, and narrow interests. It is accompanied by depression, irritability, and intellectual impairment [1] and is often associated with other neurodevelopmental disorders such as intellectual disability/global developmental delay [2], epilepsy [3], attention deficit hyperactivity disorder [4], and psychiatric behavioral disorders [5]. In recent years, several studies have demonstrated a possible correlation between 25(OH)D and ASD [6–9].

Vitamin D is a neurosteroid hormone essential for neural development [10]. As the main mechanisms in the central nervous system, nerve cell proliferation, neurotransmission, oxidative stress, and immune function are all mediated by vitamin D [11, 12]. In contrast, 25(OH)D acts as the form for vitamin D to exert its biological effects in the body. Therefore, vitamin D deficiency during pregnancy and early childhood may severely affect brain development and lead to adverse neuropsychological consequences. Severe vitamin D deficiency has been found in children with ASD and in pregnant women whose children will also develop ASD in the future. Most of the current epidemiological studies of small sample sizes (which do not reveal a causal relationship between 25(OH)D and ASD) show that 25(OH)D concentrations in children with ASD are statistically and significantly lower than in normal children [13–16]. Besides, a retrospective analysis with a sample size of 616 reached the same conclusion [17]. Notwithstanding, some epidemiological studies with larger sample sizes (which, again, do not reveal a causal relationship between 25(OH)D and ASD) indicated that 25(OH)D in children with ASD was not lower than in normal children. Gayle et al. conducted a retrospective study with a sample size of 999 people in 2019 [18], and the findings do not support the hypothesis of lower 25(OH)D being associated with a higher risk of ASD. In 2020, a meta-analysis of randomized controlled trials (RCTs) with a sample size of 349 was conducted by Bingbing Li [19], and the results showed that vitamin D supplementation appears to intensify hyperactivity rather than core symptoms or other co-existing behaviors and conditions of ASD. However, in the same year, a meta-analysis of RCTs with a sample size of 203 made by Liyao Song drew different conclusions [20], where it was proposed that vitamin D supplementation improves the typical symptoms of ASD.

In conclusion, controversy with regard to the true link between 25(OH)D and ASD has been unsettled.

Based on this, we systematically evaluated the complex relationship between 25(OH)D and ASD from the perspective of shared genetics, pleiotropy, and causality. Traditional observational epidemiological studies encounter many challenges in discovering disease etiology and causal inference, including reverse causal associations, potential confounders, minimally effective exposure factors, and multiple testing, and randomized controlled trials based on chronological order may be unethical. Since the association between genes and underlying outcomes is not confounded by common factors such as postnatal environment and socioeconomic status, and the causal time sequence is reasonable, genes acting as instrumental variables in disease association studies (i.e., MR analysis) play an important role in causal inference. Serum 25-hydroxyvitamin D (25(OH)D) reflects the combined effects of supplementation and skin production, so the level of serum 25 (OH)D is considered to be the standard measure of vitamin D status in subjects [21]. Considering that 25(OH)D and ASD may be mutually causal, a bidirectional MR analysis was implemented to test the causal relationship between 25(OH)D and ASD to avoid omission.

## Materials and methods

In this paper, the genetic correlation between 25(OH)D and ASD was assessed at first; then, pleiotropic genes associated with both phenotypes were identified using PLACO, an effective method. Finally, the biological function of the localized pleiotropic genes was explored using enrichment analysis [22], and bidirectional MR analysis was applied to investigate whether 25(OH)D is causally related to ASD.

### GWAS summary statistics

To reduce population stratification bias, all samples used in this study were obtained from European populations. 25(OH)D was taken from a genome-wide association study of 25(OH)D in 417580 Europeans [23], and ASD data were acquired respectively from a study of a unique Danish population, a genome-wide association meta-analysis of 18,381 ASD cases and 27,969 controls [24]. The 25(OH)D data download link is https://cnsgenomics.com/content/data; the ASD data download link is https://pgc.unc.edu/, data updated to June 2022. Ethical approval and informed consent were achieved for all original studies.

### Linkage disequilibrium score regression

LDSC was first used to assess shared polygenic structure between traits, where linkage disequilibrium scores (LDS) could be calculated from European samples in the 1000 Genomes Project and then used as a reference panel. For single-nucleotide polymorphism (SNP), strict quality control was implemented, by removing all non-biallelic allele SNPs, SNPs with strand-ambiguous alleles (A/T, C/G allele SNPs), SNPs with MAF < 1%, SNPs without rs numbering, duplicate rs IDs, and SNPs that were not presented or whose alleles did not match phase 3 of the 1000 Genomes Project.

### Pleiotropic analysis under composite null hypothesis

As a new way to study pleiotropic loci between complex traits, PLACO uses aggregated levels of genotype-phenotype association statistics [22]. During the process, the *Z* score’s square was calculated for each variant, and SNPs with very high *Z*^2^ (> 80) values were removed. In addition, given the potential correlation between the two diseases, the correlation matrix of *Z* was estimated, and its matrix was included in the performed analysis. Finally, the hypothesis of no pleiotropy was tested using the level-α cross-over-unit test (IUT) method, and the final pleiotropy *p*-value was determined. Functional mapping and annotation of genome-wide association studies (FUMA) were used to determine the biological function of pleiotropic [25]. The *r*^2^ threshold defining independent significant SNPs was set to 0.2, and the maximum distance between LD blocks merged into a locus was set to 500 kb. The identified locus was then mapped to nearby genes, and a series of pathway enrichment analyses were used to determine the function of the mapped genes based on the Molecular Signature Database (MSigDB) [26].

### Causal association analysis

The bidirectional MR analysis was adopted to assess the causal relationship between 25(OH)D and ASD. Notably, the MR analysis should be performed under three basic assumptions: (1) genetic variation is strongly associated with exposure, (2) genetic variation is independent of any potential confounding factors, and (3) genetic variation is not associated with outcomes except through the stated exposure pathway. This study was reported in accordance with the latest guidelines in Strengthening the Reporting of Observational Studies in Epidemiology Using Mendelian Random Assignment (STROBE- MR).

SNPs that were first subjected to Mendelian randomization studies were carefully selected, and SNPs with an *F* statistic < 10 were excluded to avoid weak instrumental bias. The instrumental variable had a PVE of 5.82% and a minimum *F* value of 10.1. A total of 123 SNPs were screened for *25(OH)D* by taking *P* < 5 × 10^−8^ and linkage disequilibrium *r*^2^ < 0.1 as screening parameters. Detailed information on IVs is provided in the supplementary material (Supplementary Material, Table 1). To verify causal relationships, MR methods and comprehensive sensitivity analysis were performed for each set of IVs. The main analysis was carried out using the IVW method based on validated instrumental variables. Weighted median and weighted-mode methods were used to validate the robustness of IVW, and MR-PRESSO’s global test and MR-Egger’s intercept term were used to identify variants with disproportionate effects and directional pleiotropy. In addition, we used scatter plots and funnel plots to assess the robustness of the associations. Besides, the IVW method [27], the weighted median method [28], and the MR-Egger method [29] were respectively used to investigate the association between 25(OH)D and ASD. These methods are all commonly used in MR studies, with each having its own strengths and weaknesses in terms of consistency, test validity of causal effect estimates, and untestable assumptions. Besides, their effectiveness in finding causal effects varies. Research has indicated that the IVW method is more effective in finding causal effects than the weighted median method and MR-Egger analysis. However, this method relies on strong assumptions, so it may have an increased type I error rate and bias in the estimation of causal effects. The MR-Egger method was more influenced by the Inside assumptions, and when the inside assumptions were met, the defects of the IVW method can be well controlled, but its effectiveness can be restricted when the inside assumptions were not met [28]. The weighted median method is not distinctive compared to the other two methods, but it is far superior when the inside assumptions are violated, provided that the number of invalid instrumental variables is not too large. The MR-PRESSO method [30] is a widely used method for testing horizontal multiplicity of effects, and it has three core components: (1) the MR-PRESSO global test to detect the presence of horizontal pleiotropy, (2) the MR-PRESSO outlier test to remove abnormal SNPs (outliers) and to estimate the corrected SNPs (outliers) and estimate the corrected result (which removes horizontal pleiotropy), and (3) the “MR-PRESSO distortion test” to test for differences between the pre-corrected and post-corrected results.

The above statistical analyses were performed in the R3.5.3 software, and MR analysis was conducted using the Mendelian Randomization package [31]. MR-PRESSO was made using the MRPRESSO package [31]. Multiple validity analysis under the compound null hypothesis was implemented using PLACO software [32]. Genetic correlation analysis was accomplished using chain disequilibrium score regression (LDSC) [33].

## Results

### Genetic correlation between 25(OH)D and ASD

In this study, we observed a negative correlation between genetically determined 25(OH)D and ASD risk *(r*_g_ = − 0.227, *P <* 0.05), and this suggests a potential shared mechanism between 25(OH)D and ASD.

### Pleiotropic gene loci identified for 25(OH)D and ASD with PLACO

**Fig. 1:**
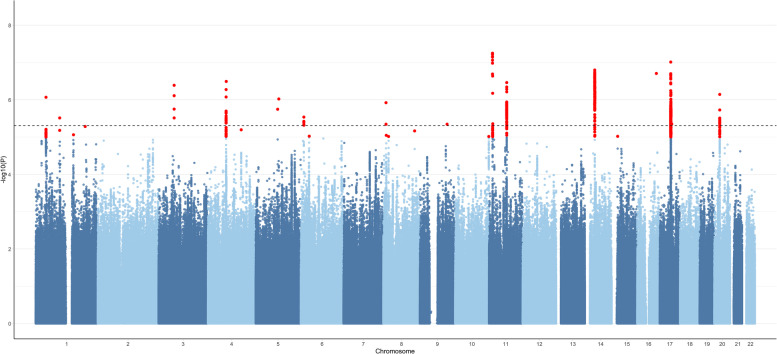
Manhattan plot of the PLACO results. In the figure, the horizontal line represents the significance of 5E-8. *r*^2^ threshold to define independent significant SNPs was set to 0.2, and the maximum distance between LD blocks to merge into a locus was set to 500 kb; The *r*^2^ threshold to define independent significant SNPs was set to 0.2, and maximum distance between LD blocks to merge into a locus was set to 500 kb.

The pleiotropy analysis identified 20 independent pleiotropic loci (*P* < 5 × 10^−6^), which were mapped to 24 pleiotropic genes, as indicated in Fig. 1 and Table 1.

**Table 1:** Details of SNPs and pleiotropic mapped genes

**SNP**	**Pleiotropic genes**
rs56044892	CRHR1
rs1222064	GSTM3P
rs62243489	LOC100132258
rs13108630	LOC644253
rs72653968	LOC644620
rs12653396	NPFFR2
rs2635182	SEC23A
rs932345	C17orf69
rs1986735	C6orf105
rs7951630	CADPS
rs4373971	GC
rs117702603	KIAA1267
rs3814731	LOC100129319
rs55737943	LOC100132074
rs150157011	LOC283547
rs1631850	LOC391810
rs2141298	LOC645323
rs199535	LOC729977
rs17644409	NADSYN1
rs2424369	NKX2-4
	NSF
	PDE3B
	SPON1
	SPOP

Later, we further performed enrichment analysis for pleiotropic genes and found that they were enriched through three pathways, i.e., regulation of secretion by cell, cellular response to hormone stimulus, and intracellular protein-containing complex. In the process, no significant enrichment of pleiotropic genes was observed in different tissues, but differential expression of GC gene appeared in the liver, pancreas, and stomach. In addition, two of the top 3 enriched tissues were both brain tissues. See supplementary material for details (Supplementary Material, Figures 1-3).

### Causal association between 25(OH)D and ASD

In a Mendelian randomization analysis on VD–ASD correlation, the IVW method showed OR = 0.941 (0.796, 1.112) and did not show a causal relationship between 25(OH)D and ASD.

### Causal association between ASD and 25(OH)D

A reverse MR analysis was also performed simultaneously in this study, again taking *P* < 5 × 10^−8^ and chain imbalance *r*^2^ < 0.1 as screening parameters, and a total of 1 SNP was screened. Only the IVW method and a fixed model were respectively conducted and taken, yielding an OR = 1.042 (0.930, 1.169). No statistical significance was found either.

**Fig. 2:**
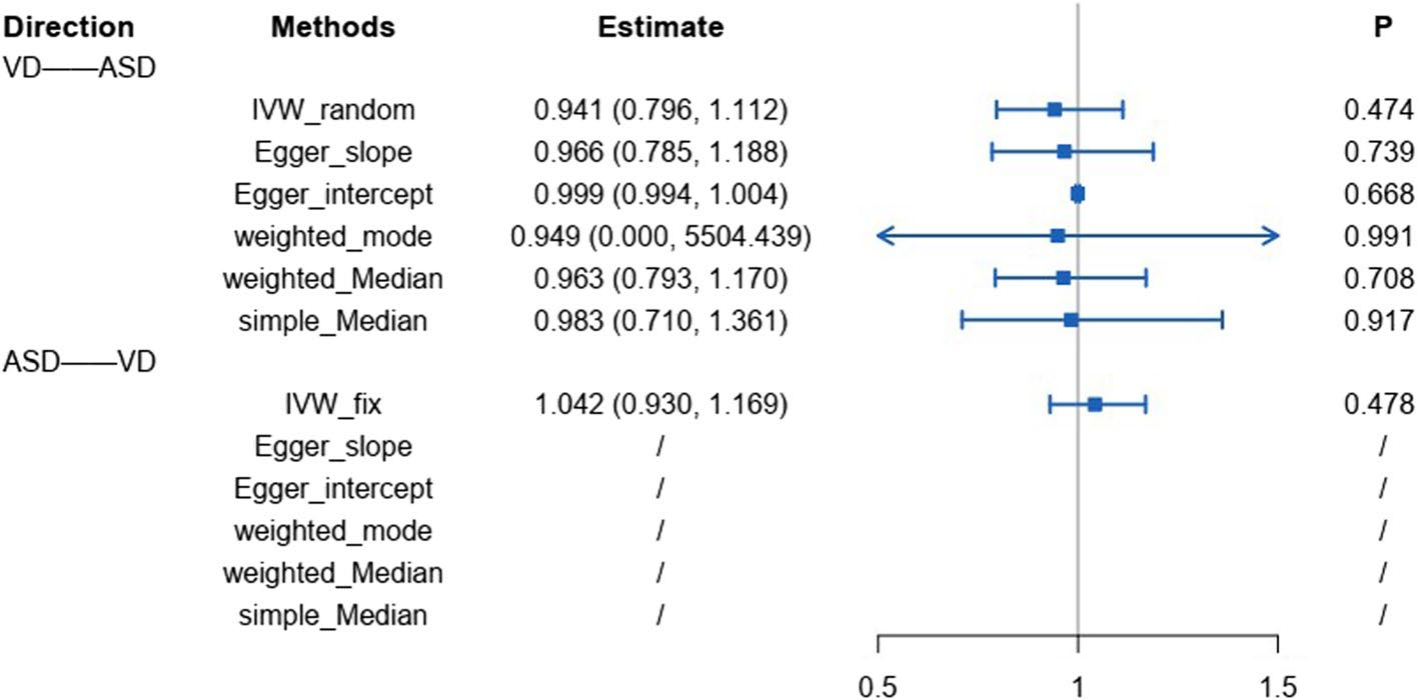
Details of bidirectional MR

In the Mendelian randomization analysis on VD–ASD correlation, the accuracy of IVW was subsequently verified using the weighted median and weighted-mode methods in order to ensure that the results were convincing. See Fig. 2 for details.

**Fig. 3:**
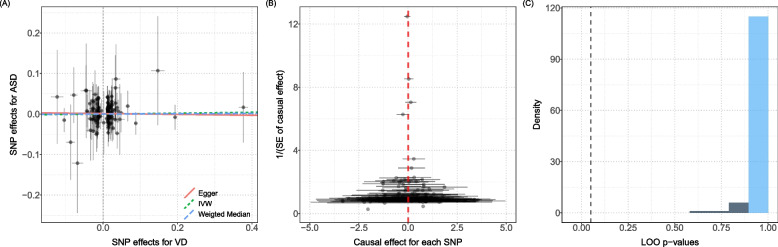
**A** In the figure, the *x*-axis (SD units) represents the effect of SNP on 25(0H) D and the *y*-axis (log OR) represents the effect of SNP on ASD. Each black dot represents an individual SNP, and the line segment represents the 95% CI. The slopes of the 3 straight lines correspond to the causal estimates of the 3 MR methods, green for the IVW method, red for the MR-Egger method, and light blue for the weighted median method. **B** In the figure, the black dots represent the log OR of the ASD increase in standard deviation (SD) in 25(OH)D. The OR was generated using each SNP as a separate instrumental variable. **C** No single SNP significantly affects the conclusions, and the results are stable

Both MR-PRESSO’s global test and MR-Egger’s intercept term were used to identify variants with disproportionate effects and directional pleiotropy. Besides, scatter plots and funnel plots were adopted to assess the robustness of the association. The scatter plot indicated that there were no multi-effect outliers that affected causal inference; the funnel plot showed that the causal estimates were evenly distributed on both sides of the effect estimates and the results were robust. Moreover, LOOCV results demonstrated that, after excluding one instrumental variable at a time for analysis, the frequency distribution plot found no SNPs interfere with causal inference. See Fig. 3.

## Discussion

This study is the first to combine three methods, LDSC, pleiotropy analysis, and bidirectional MR to comprehensively assess the relationship between 25(OH)D and ASD. Based on large-scale GWAS summary statistics, we first used LDSC to find evidence of negative shared genetic overlap between 25(OH)D and ASD. Then, we used PLACO to identify 20 independent pleiotropic loci (*P* < 5 × 10^−6^), which are matched to 24 pleiotropic genes. This is followed by further enrichment analysis. Finally, we used bidirectional MR to analyze whether there was a causal relationship between 25(OH)D and ASD, and the result was negative.

A retrospective study carried out in 2017 included 308 children with ASD and an equal number of healthy control children. The results revealed that vitamin D deficiency was considerably more common among autistic children as compared to healthy children. As epidemiological studies were further conducted, sufficient papers were published for meta-analysis and systematic evaluation, and in 2020, Zuqun Wang included 24 case-control studies [34], concluding that vitamin D concentrations were significantly lower in children and adolescents with ASD than in controls. Besides, a meta-analysis of maternal and neonatal vitamin D was also made in the above said research, which found a decreasing trend in early vitamin D concentrations in the ASD group. The results of LDSC in this study also demonstrated a negative and overlapping genetic correlation between 25(OH)D and ASD. This is consistent with the conclusions of the mainstream epidemiological view.

By using PLACO, it was found that the main enrichment appeared in the three pathways, i.e., regulation of secretion by cell, cellular response to hormone stimulus, and intracellular protein-containing complex. Previous similar literature has demonstrated that related pathways are involved in the genesis of ASD [31], and other research reports also indicated that the Wnt pathway was clearly involved in 25(OH)D and ASD, and that one of the functions of 25(OH)D was to maintain the expression of dickopf-1 (DKK-1) [32], which acted as an inhibitor of the Wnt/β-catenin signaling pathway [33–35]. The GC gene was of great interest to us, which was found in the liver, pancreas, and stomach. Different tissue enrichment analyses have found that it is mainly concentrated in the brain, which could also suggest a possible role of the GC gene in 25(OH)D and ASD [36, 37]. In a case-control study of autism spectrum disorders (ASD) using a genome-wide association study on total 25(OH)D of maternal mid-gestational, serum-derived and neonatal blood spot-derived [36], a fetal locus in the GC gene was found to be significantly associated with neonatal vitamin D levels, since it encodes a binding protein for vitamin D transport and function.

Several possible mechanisms may explain the relationship between 25(OH)D and ASD. Vitamin D deficiency in the developing brain affects many important processes, for example, the synthesis of neurotransmitters including NO, cellular differentiation, Ca^2+^ signaling, and antioxidant activity [38]. In the central nervous system, NO acts as both a neurotransmitter and a neuromodulator. With low concentrations, NO exerts physiological effects on nerve cells and vascular cells; with high concentrations, NO is toxic and may lead to cell death, which is implicated in the pathogenesis of many neurological disorders such as stroke and neurodegenerative diseases, whereas 25(OH)D is closely related to NO production, and they regulate each other’s concentrations [39]. Furthermore, 25(OH)D deficiency may contribute to autism through alterations in Ca^2+^ signaling processes that control brain development [40]. One of the main functions of 25(OH)D is to maintain low resting levels of Ca^2+^ and ROS. Genetic analysis of a previous study revealed that the Ca^2+^ signaling pathway plays a major role in autism development, which may be caused by the control action of precise Ca^2+^ transients on brain development [41]. These spontaneous Ca^2+^ transients occurring during brain development may be produced by subtle changes in Ca^2+^ dynamics due to 25(OH)D deficiency. It has also been suggested that 25(OH)D deficiency leads to increased expression of CaV1.2 and CaV1.3 channels [42], which in turn allows abnormal Ca^2+^ levels and affects neurodevelopmental processes [43].

The mechanism of 25(OH)D production is linked to other factors that further influence ASD development, so it is important to investigate the direct causal relationship between 25(OH)D and ASD. As a result, after identifying the definitive evidence on a link between 25(OH)D and ASD, we attempted to further investigate it in depth.

In previous studies, partial cause-to-effect studies with high-level evidence-based medical evidence concluded that the causal relationship between 25(OH)D and ASD is unclear. Kerley found in a double-blind, randomized, and placebo controlled trial developed in 2017 that vitamin D supplementation had no effect on the primary outcome with limited and inconsistent effects in children with ASD [44], whereafter a cohort study with a larger sample size emerged, in which 3852 children were included. During the observation period (mean follow-up of 2.5 years), 41 children were diagnosed with an ASD. No association was found between 25(OH) D concentrations and ASD in both unadjusted and adjusted models. An association between vitamin D supplementation in early childhood and ASD was also not identified. Vitamin D in early childhood may not be associated with physician diagnostic events for ASD. Therefore, as is known to all, epidemiological surveys with high levels of evidence remain unclear on the causal relationship between 25(OH)D and ASD, and most tend to believe that there is no causal relationship. In current studies, the IVW method does not show a causal relationship between 25(OH)D and ASD based on a bidirectional MR analysis with OR = 0.94 (0.796, 1.112); in the opposite Mendelian randomization analysis, the IVW method has concluded OR = 1.042 (0.930, 1.169), which still does not indicate a causal relationship. The result is consistent with the findings of most high-quality epidemiological studies. In conclusion, there is reason for us to believe that 25(OH)D and ASD may not be causally related, but this result needs to be viewed with caution. Although our study has not found strong evidence of causality between 25(OH)D and ASD, it cannot be definitely excluded. The possible contribution of 25(OH)D single-nucleotide pleiotropies’ genetic effect is to increase the risks of ASD. In regard to this, further validation using large, multicenter, double-blind, randomized and controlled trials is required.

This study also has some limitations. On one hand, the data applied in this study were only available from the GWAS summary data as individual data were not available. This limits stratification and thus the assessment of the non-linear relationship between 25(OH)D and ASD. On the other hand, the data included in the study were all European sources, and the findings were somewhat limited. And the GWAS for 25OH-vitamin D comes from a different population as the GWAS on ASD, which is from a unique and homogenous Danish population.

## Conclusion

The present study provides evidence for a shared genetic overlap between 25(OH)D and ASD. However, bidirectional MR analysis do not show a definite causal relationship between 25(OH)D and ASD, so the results need to be treated with caution. Larger, multicentered, and double-blinded RCTs are needed to validate our results.

## Supplementary Information

**Additional file 1: Supplementary Material, Table 1.** DetailedAQ information on IVs.

**Additional file 2: Supplementary Material, Figures 1–3.** Enrichment analysis for pleiotropic genes.

## Data Availability

The data used to support the findings of this study are included with in the article.
