# Supplementary figures and images for: 25(OH)Vitamin D and autism spectrum disorder: genetic overlap and causality
Source: Genes Nutr. 2023 Apr 26;18:8. doi: 10.1186/s12263-023-00727-0 (PMC10134540; doi:10.1186/s12263-023-00727-0)

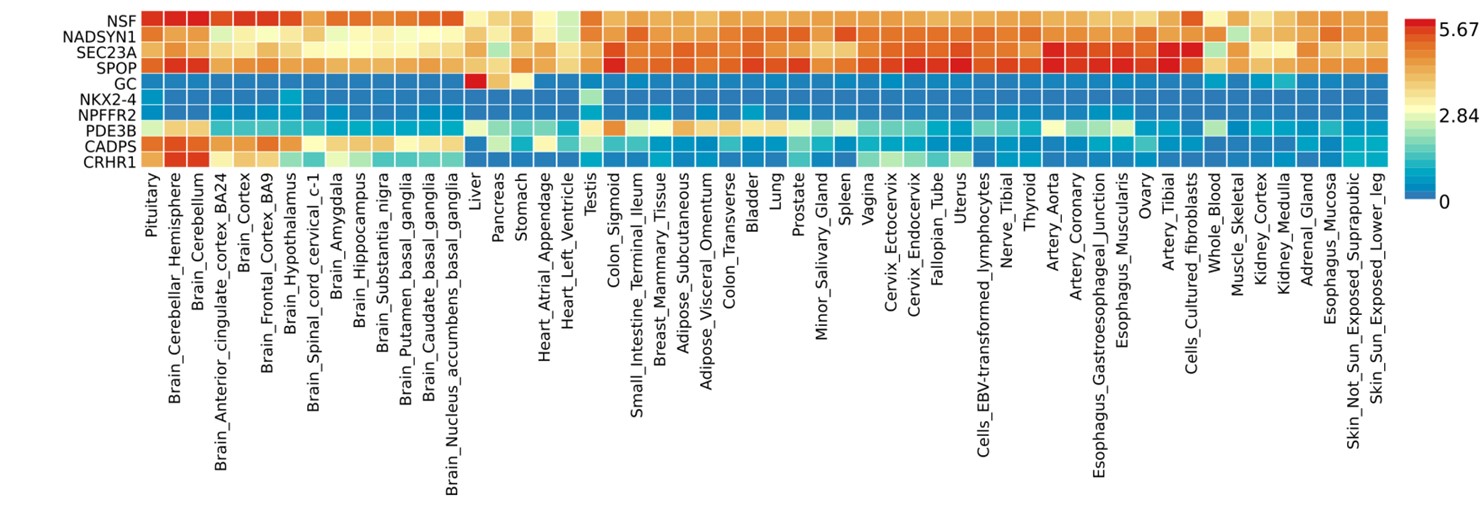

Supplement: Supplementary file 2 — Additional file 2: Supplementary Material, Figures 1–3. Enrichment analysis for pleiotropic genes. [file 12263_2023_727_MOESM2_ESM.zip › Supplementary Figure 1.jpg]

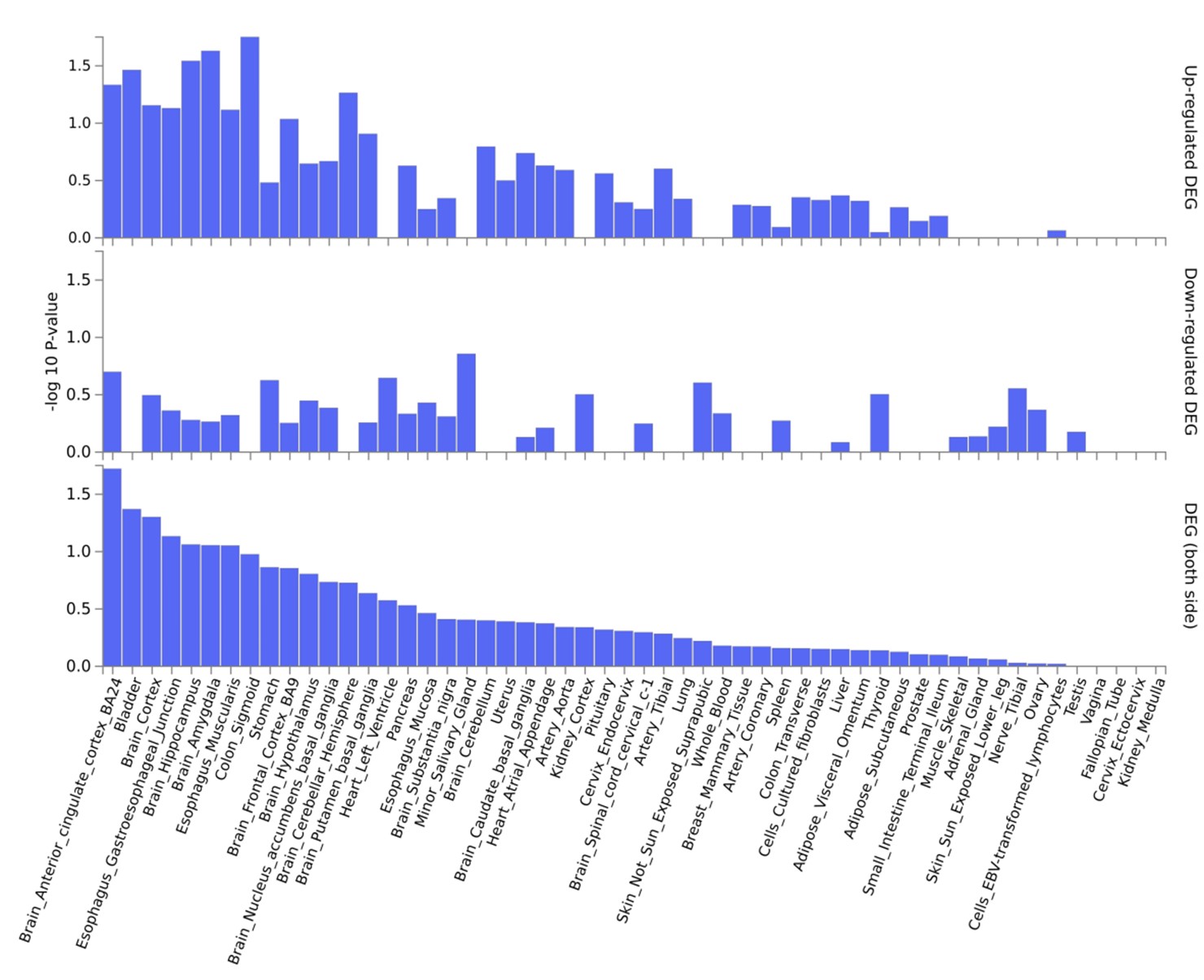

Supplement: Supplementary file 2 — Additional file 2: Supplementary Material, Figures 1–3. Enrichment analysis for pleiotropic genes. [file 12263_2023_727_MOESM2_ESM.zip › Supplementary Figure 2.jpg]

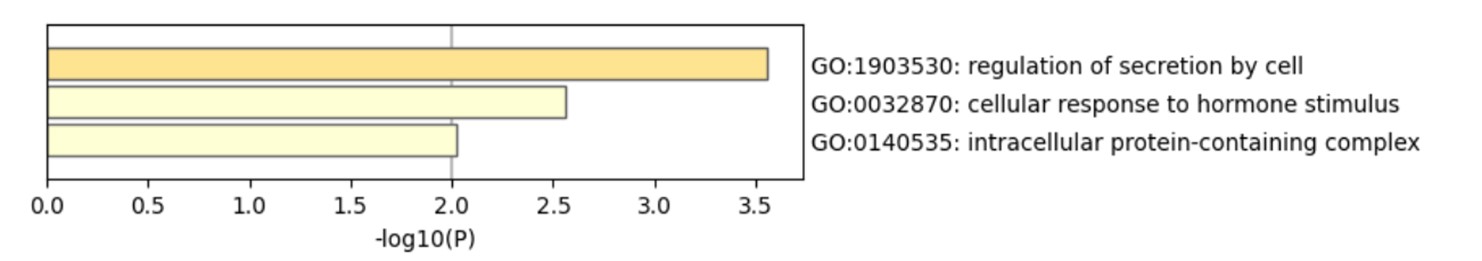

Supplement: Supplementary file 2 — Additional file 2: Supplementary Material, Figures 1–3. Enrichment analysis for pleiotropic genes. [file 12263_2023_727_MOESM2_ESM.zip › Supplementary Figure 3.jpg]
